# Structures of the hydrolase domain of zebrafish 10-formyltetrahydrofolate dehydrogenase and its complexes reveal a complete set of key residues for hydrolysis and product inhibition

**DOI:** 10.1107/S1399004715002928

**Published:** 2015-03-27

**Authors:** Chien-Chih Lin, Phimonphan Chuankhayan, Wen-Ni Chang, Tseng-Ting Kao, Hong-Hsiang Guan, Hoong-Kun Fun, Atsushi Nakagawa, Tzu-Fun Fu, Chun-Jung Chen

**Affiliations:** a Life Science Group, Scientific Research Division, National Synchrotron Radiation Research Center, Hsinchu 30076, Taiwan; b Department of Medical Laboratory Science and Biotechnology, National Cheng Kung University, Tainan City 701, Taiwan; c Department of Pharmaceutical Chemistry, College of Pharmacy, King Saud University, Riyadh 11451, Saudi Arabia; d X-ray Crystallography Unit, School of Physics, Universiti Sains Malaysia, 11800 USM Penang, Malaysia; e Institute for Protein Research, Osaka University, Suita, Osaka 565-0871, Japan; f Institute of Biotechnology and University Center for Bioscience and Biotechnology, National Cheng Kung University, Tainan City 701, Taiwan; g Department of Physics, National Tsing Hua University, Hsinchu 30043, Taiwan

**Keywords:** 10-formyltetrahydrofolate dehydrogenase, zebrafish

## Abstract

10-Formyltetrahydrofolate dehydrogenase (FDH), which is composed of a small N-terminal domain (Nt-FDH) and a large C-terminal domain, is an abundant folate enzyme in the liver and converts 10-formyltetrahydrofolate (10-FTHF) to tetrahydrofolate (THF) and CO_2_. Nt-FDH alone possesses a hydrolase activity, which converts 10-FTHF to THF and formate in the presence of β-mercaptoethanol. To elucidate the catalytic mechanism of Nt-FDH, crystal structures of apo-form zNt-FDH from zebrafish and its complexes with the substrate analogue 10-formyl-5,8-dideazafolate (10-FDDF) and with the products THF and formate have been determined. The structures reveal that the conformations of three loops (residues 86–90, 135–143 and 200–203) are altered upon ligand (10-FDDF or THF) binding in the active site. The orientations and geometries of key residues, including Phe89, His106, Arg114, Asp142 and Tyr200, are adjusted for substrate binding and product release during catalysis. Among them, Tyr200 is especially crucial for product release. An additional potential THF binding site is identified in the cavity between two zNt-FDH molecules, which might contribute to the properties of product inhibition and THF storage reported for FDH. Together with mutagenesis studies and activity assays, the structures of zNt-FDH and its complexes provide a coherent picture of the active site and a potential THF binding site of zNt-FDH along with the substrate and product specificity, lending new insights into the molecular mechanism underlying the enzymatic properties of Nt-FDH.

## Abstract 2

Structures of the hydrolase domain of 10-formyltetrahydrofolate dehydrogenase from zebrafish and its complexes are reported.

## 1. Introduction   

Folate (vitamin B_9_) is an essential nutrient for embryonic development and the maintaince of proper physiological functions. Folate serves as a one-carbon carrier in cells and is heavily involved in many fundamental metabolic reactions, including the biosynthesis of purine, choline, amino acids and neurotransmitters and methyl-group biogenesis. Therefore, folate and folate-requiring one-carbon metabolic (OCM) enzymes are crucial for cell proliferation and epigenetic control of gene activity. Among the enzymes participating in OCM, 10-formyltetrahydrofolate dehydrogenase (FDH; ALDH1L1; EC 1.5.1.6) converts 10-formyltetrahydrofolate (10-FTHF) to tetrahydrofolate (THF) and CO_2_, the final step in methanol detoxification, using NADP^+^ as the hydride-ion receptor. 10-FTHF participates in purine biosynthesis in the purine cycle, where two one-carbon units are transferred from 10-FTHF to form the purine ring. FDH binds tightly and stabilizes its own product THF, a very unstable and easily oxidized vitamin (Kim *et al.*, 1996 ▶; Fu *et al.*, 1999 ▶). FDH is abundant in liver and kidney, the two primary organs for folate metabolism, and comprises approximately 1% of the total soluble protein in liver (Krupenko & Oleinik, 2002 ▶; Min *et al.*, 1988 ▶). Currently, the biological significance of FDH under normal physiological conditions is not completely understood. Several possibilities have been proposed, including modulation of the intracellular levels of the one-carbon unit and THF (Anguera *et al.*, 2006 ▶), the regulation of formate oxidation (McMartin *et al.*, 1977 ▶) and serving as a storage reservoir for THF (Champion *et al.*, 1994 ▶; Neymeyer *et al.*, 1997 ▶). Functions independent of the FDH catalytic activity have also been revealed in several recent reports. Despite being considered to be a housekeeping metabolic enzyme, FDH has been shown to modulate the cell cycle, to possess suppressor effects and to be downregulated in tumour tissues (Krupenko & Oleinik, 2002 ▶). This anti-apoptotic activity endows FDH with the potential to serve as a chemotherapeutic target. In addition, FDH possesses antioxidative activity, which is crucial for embryo­genesis and modulating intracellular oxidative stress (Hsiao *et al.*, 2014 ▶; Chang *et al.*, 2014 ▶). These observations add more emphasis to the importance of understanding the structural and catalytic properties of FDH.

Most FDHs studied to date consist of four identical subunits of approximately 100 kDa (Schirch *et al.*, 1994 ▶; Krupenko *et al.*, 1997 ▶). Each subunit comprises two independent folded domains connected by an intermediate domain. The small N-terminal domain only exhibits an NADP^+^-independent 10-FTHF hydrolase activity. The large C-terminal domain of mammalian FDH possesses an NADP^+^-dependent aldehyde dehydrogenase activity, which, however, is not detected in zebrafish FDH (zFDH; Krupenko, 2009 ▶; Chang *et al.*, 2010 ▶). Exhibition of the 10-FTHF dehydrogenase activity requires the proper conformational arrangement of both the N- and C-terminal domains and the presence of 4′-phosphopante­theine (4′-PP; Chang *et al.*, 2010 ▶; Donato *et al.*, 2007 ▶). This 4′-PP is bound to Ser354 of the intermediate domain in rat FDH (Ser355 in zFDH) and is proposed to transfer the formyl group between the catalytic hydrolase and NADP^+^-dependent dehydrogenase domains of FDH (Cook *et al.*, 1991 ▶; Schirch *et al.*, 1994 ▶; Krupenko, 2009 ▶). FDH purified from rabbit liver contains tightly bound THF polyglutamates (*K*_d_ = 15 n*M*) and exhibits product inhibition (Fu *et al.*, 1999 ▶; Kim *et al.*, 1996 ▶). The N-terminal hydrolase domain of FDH (Nt-FDH) binds folate and is homologous to glycinamide ribonucleotide formyltransferase (GART) and methionyl-tRNA formyltransferase (FMT), enzymes that are crucial for *de novo* purine biosynthesis and translation initiation, respectively. The crystal structures of the N- and C-terminal domains of mammalian FDH have been determined separately (Kursula *et al.*, 2006 ▶; Chumanevich *et al.*, 2004 ▶; Tsybovsky *et al.*, 2007 ▶). However, no crystal structure of full-length FDH has ever been documented.

Zebrafish is an animal model that has recently risen in prominence in research in developmental biology, disease pathomechanisms and drug screening (Kari *et al.*, 2007 ▶). Zebrafish FDH highly resembles its mammalian orthologues both structurally and functionally, and is also a homotetramer. Each monomer contains 903 amino-acid residues and comprises an N-terminal domain (amino acids 1–310) and a C-terminal domain (amino acids 420–903) connected by an intermediate domain (amino acids 311–419). The intermediate domain is an ACP-like domain (Chang *et al.*, 2010 ▶, 2014 ▶). In this study, we determined crystal structures of zebrafish wild-type apo Nt-FDH (zNt-FDH) and its ternary complexes with the substrate 10-formyl-5,8-dideazafolate (10-FDDF) and the products tetrahydrofolate (THF) and formate. The complete structures of the substrate and the product in the active site are revealed, as well as the key residues involved in substrate binding and recognition. An additional potential THF binding site is observed in the crystal structure and its probable physiological significance is discussed. The catalytic mechanism underlying the hydrolase reaction is also suggested based on comparative analysis of the zNt-FDH Y200A mutant and the structural alterations associated with substrate and product binding.

## 2. Experimental procedures   

### 2.1. Protein preparation and site-directed mutagenesis   

The protein expression and purification of zNt-FDH have been described previously (Chang *et al.*, 2010 ▶). *Escherichia coli* cells containing the zNt-FDH/pET-43.1a plasmid were grown at 37°C in LB broth with 100 µg ml^−1^ ampicillin. After a brief heat shock by incubating at 42°C for 15 min, FDH was induced at 25°C with 70 µ*M* isopropyl β-d-1-thiogalacto­pyranoside (IPTG) for 3 h when bacterial growth reached the log phase. A nickel column was used to purify zNt-FDH following the manufacturer’s protocol. zNt-FDH eluted at 20 m*M* Tris–HCl pH 7.5, 300 m*M* NaCl, 150 m*M* imidazole. The protein was further dialyzed into a buffer consisting of 300 m*M* NaCl, 20 m*M* Tris–HCl pH 7.5 to remove imidazole. Site-directed mutagenesis was performed using the QuikChange method (Stratagene). The synthetic oligo­nucleotides used for mutagenesis were as follows. F89A: forward primer, 5′-TTC TAC CTT TCT GCT CTC AGG CCA TCC CAA TGG AAG TGA TAG-3′; reverse primer, 5′-CTA TCA CTT CCA TTG GGA TGG CCT GAG AGC AGA AAG GTA GAA-3. R114A: forward primer, 5′-GCT GCC CAG ACA CGC AGG TGC CTC TGC C-3′; reverse primer, 5′-GGC AGA GGC ACC TGC GTG TCT GGG CAG C-3′. Y200A: forward primer, 5′-GAA GGA GCC ACA GCT GAG TGT ATT C-3′; reverse primer, 5′-GAA TAC ACT CAG CTG TGG CTC CTT C-3. K205A: forward primer, 5′-GGA GCC ACA TAT GAG TGT ATT CAG GCG AAA GAA AAT TCA AAG ATT GAC TG-3′; reverse primer, 5′-CAG TCA ATC TTT GAA TTT TCT TTC GCC TGA ATA CAC TCA TAT GTG GCT CC-3′. The basic procedure involved PCR amplification using the plasmid zNt-FDH/pET-43.1a as the template and two synthetic oligonucleotides containing the desired mutation as the primers. Successful mutagenesis was confirmed by nucleotide sequencing. The purification procedures for the mutant enzymes were the same as that for the wild-type enzyme.

### 2.2. Enzymatic activity assay   

FDH catalyzes the conversion of 10-FTHF to THF and CO_2_ using NADP^+^ as a cofactor. 10-FTHF and THF were generous gifts from Dr Rudolf Moser (Merck Eprova AG, Switzerland). The hydrolase activities of zNt-FDH and its mutants were determined by monitoring the rate of tetrahydrofolate formation (OD at 298 nm) following the protocols described previously for determining the apparent *k*_cat_ and *K*_m_ values of FDH (Schirch *et al.*, 1994 ▶). Briefly, the reaction was initiated by adding zNt-FDH or mutants (15 µg) to a reaction mixture (0.8 ml) containing 20 µ*M* 10-FTHF, 0.5 µ*M* zebrafish cSHMT and 0.1 m*M* β-mercaptoethanol. Adding cSHMT to the reaction mixture relieved the product inhibition. All assays were performed in a 1 cm cuvette at 30°C.

### 2.3. Crystallization of complexes of wild-type zNt-FDH and the mutant   

All crystallizations were undertaken using the hanging-drop vapour-diffusion method in a 48-well ADX plate (Hampton Research) at 291 K. 1 µl protein solution (∼10 mg ml^−1^ in 300 m*M* NaCl, 20 m*M* Tris–HCl pH 7.5) was mixed with 1 µl reservoir solution and equilibrated against 120 µl reservoir solution. Crystals of wild-type zNt-FDH (zNt-FDH_WT) and the zNt-FDH Y200A mutant (zNt-FDH_Y200A) were grown with a crystallization solution consisting of 0.1–0.2 *M* bis-tris pH 5.5, 25–29%(*w*/*v*) PEG 3350. Crystals of the complexes of zNt-FDH_WT with 10-FDDF, THF and sodium formate as well as the complex of zNt-FDH_Y200A with 10-FDDF were prepared by soaking single crystals of apo zNt-FDH_WT or zNt-FDH_Y200A (∼0.2 × 0.2 × 0.1 mm) in the crystallization solution with ligands (10 m*M* 10-FDDF, THF or sodium formate, respectively) for 15 min and were flash-cooled in liquid nitrogen for the X-ray diffraction experiment. 10-FDDF was a generous gift from Dr Verne Schirch (Virginia Commonwealth University, USA).

### 2.4. Data collection and processing   

X-ray diffraction data from crystals of wild-type zNt-FDH were first collected at 110 K using a CCD detector (Q210R, ADSC) at a wavelength of 1.0 Å on the Taiwan-contracted beamline BL12B2 at SPring-8, Japan. 180° of rotation was measured with 1.0° oscillation, an exposure duration of 15 s and a crystal-to-detector distance of 180 mm at 110 K using a cryosystem (X-Stream, Rigaku/MSC). All other X-ray diffraction data were collected at 110 K on beamlines BL13B1, BL13C1 and BL15A1 equipped with CCD detectors (ADSC Q315 and Rayonix MX300HE) at the National Synchrotron Radiation Research Center (NSRRC), Taiwan and on BL12B2 and BL44XU with a CCD detector (Rayonix MX225HE) at SPring-8. All data sets were indexed, integrated and scaled using *HKL*-2000 (Otwinowski & Minor, 1997 ▶). Details of the data statistics are given in Table 1 ▶.

### 2.5. Determination and refinement of structures   

The structure of wild-type zNt-FDH was solved by molecular replacement using the structure of Nt-FDH from rat (PDB entry 1s3i) as the search model (sequence identity 73%; Chumanevich *et al.*, 2004 ▶). Throughout the refinement, a random selection (5%) of the data was set aside as a ‘free data set’ and the model was refined against the remaining data as a working data set (Brünger, 1992 ▶). Model building and refinement were performed using *Coot*, *REFMAC*5 and *PHENIX* (Emsley *et al.*, 2010 ▶; Vagin *et al.*, 2004 ▶; Adams *et al.*, 2010 ▶). Several cycles of model building using *Coot* and refinement with the individual atomic displacement parameters, TLS (translation, libration, screw) and target weight options were used to improve the quality and completeness of the structures. The refinement proceeded through another cycle of individual *B*-factor refinement and water assignment with *Coot* and *PHENIX*. The refinement converged to a final *R*_work_ of 18.0% (*R*_free_ of 20.7%) at a resolution of 1.75 Å. The structures of the wild-type complexes and the apo form and complex of the Y200A mutant were determined by molecular replacement using the determined wild-type structure as the search model with *MOLREP* (Vagin & Teplyakov, 2010 ▶). The structures were refined using procedures similar to those described above. The correctness of the stereochemistry of the models was verified with *MolProbity* (Chen *et al.*, 2010 ▶). The calculations of root-mean-square deviations from ideality for bonds, angles and dihedral and improper angles showed satisfactory stereochemistry. All crystallographic data and refinement statistics are summarized in Table 1 ▶. The structures have been deposited in the PDB (http://www.wwpdb.org) as entries 4ts4, 4tt8, 4qpd, 4qpc, 4tts and 4r8v. The figures were generated using *PyMOL* (http://www.pymol.org). Table 1 ▶ shows details of the refinement statistics.

### 2.6. Dynamic light-scattering analysis   

The effect of THF on the dimerization of zNt-FDH was investigated using dynamic light scattering (DLS; DynaPro NanoStar from Wyatt Technology). For DLS analysis, purified zNt-FDH was concentrated to a final concentration of 2.0 mg ml^−1^ in the same buffer as the protein buffer used for crystallization (20 m*M* Tris–HCl pH 7.5, 300 m*M* NaCl). The ligand THF (1 µ*M*–1 m*M*) was added to the protein solution to prepare mixtures at various molar ratios and was incubated at room temperature for 20–30 min before measurements. 10-FDDF was added to the protein solution under the same conditions as a control. The DLS measurements were performed at 25°C. Data were evaluated with an acquisition time of 5 s; the hydrodynamic radius (*R*_h_) was estimated from the average over ten acquisitions for each sample.

### 2.7. Western blot analysis   

Protein–ligand samples at various molar ratios (1:0 to 1:10 zNt-FDH:THF) containing 12 µg zNt-FDH in buffer consisting of 20 m*M* Tris–HCl pH 7.5, 300 m*M* NaCl with a total volume of 6 µl were mixed with 2 µl 4× sample-loading buffer (8% SDS, 1.2 *M* β-mercaptoethanol, 40% glycerol, 0.4% bromophenol blue, 250 m*M* Tris–HCl pH 6.8) and separated on 12% Tris–glycine SDS–PAGE. Separated proteins on the gels were electrophoretically transferred onto a nitrocellulose membrane at 85 mA for 30 min. The blotted membrane was blocked with skimmed milk (5%) in PBS for 45–60 min. After the membrane had been washed with PBS containing 0.05% Tween 20 (PBS-T buffer), the anti-His antibody (HRP; 2.57 mg ml^−1^; Genetex, Irvine, California, USA) was added (1:5000 dilution) in PBS-T buffer and incubated for 60 min. The bound antibodies were detected and stained with 3,3′-diaminobenzidine (DAB; ACROS Organics).

## 3. Results   

### 3.1. The overall structure of zNt-FDH   

**Figure 1:**
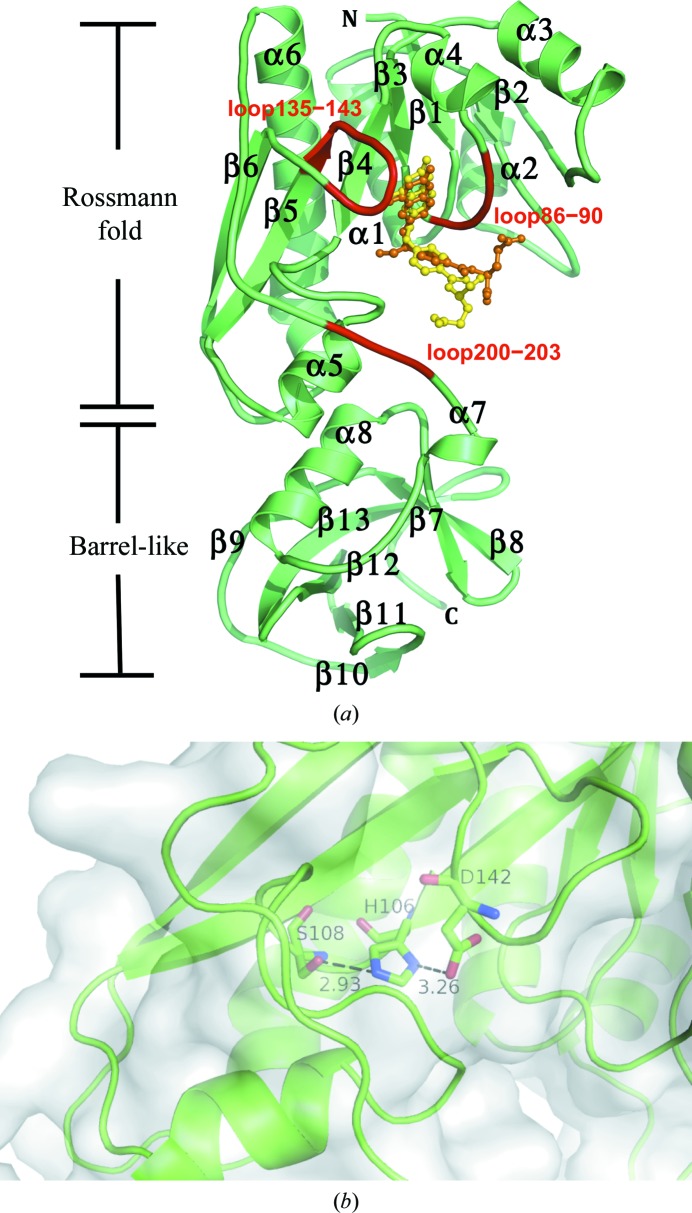
The crystal structure and active site of zNt-FDH. (*a*) The overall structure of the zNt-FDH–THF complex is divided into two subdomains, N-­terminal (subdomain 1) and C-terminal (subdomain 2), connected by a long stretch of polypeptide chain. Subdomain 1 contains a six-stranded sheet (parallel β1–β4 and antiparallel β5–β6) and six α-helices. This folate-binding domain constitutes a Rossmann fold, whereas subdomain 2 represents a slightly open β-barrel. Three loops (coloured red) surround the active-site pocket, with superimposed 10-FDDF (orange) and THF (yellow) molecules shown in ball-and-stick representation at the binding position. (*b*) The molecular surface of apo zNt-FDH is coloured grey and the residues of the catalytic triad, His106, Ser108 and Asp142, are shown in stick representation (green). Hydrogen bonds are presented as black dotted lines with distances indicated.

**Figure 2:**
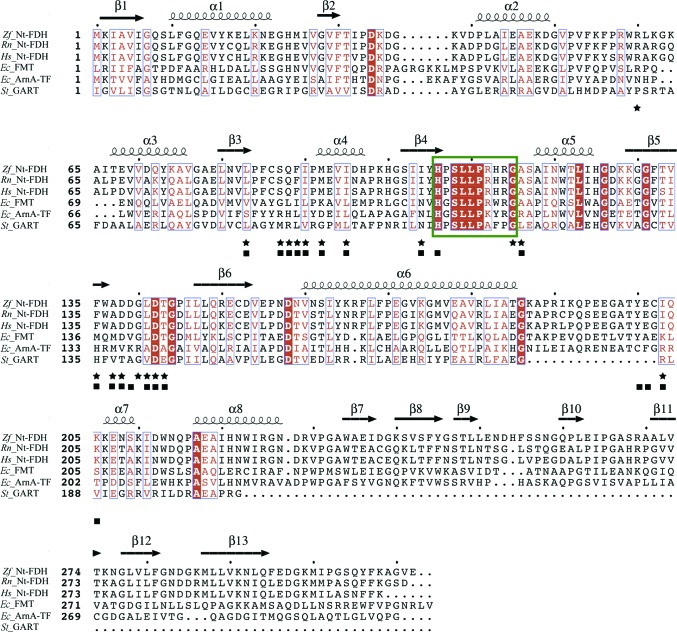
Multiple sequence alignment of related hydrolase domains of 10-formyltetrahydrofolate dehydrogenase and other folate-utilizing proteins. The sequences of various folate-utilizing proteins are aligned with that of zNt-FDH (*Zf*_Nt-FDH) to ascertain the similarities of their amino-acid sequences. The aligned proteins include Nt-FDH from *Rattus norvegicus* (*Rn*_Nt-FDH; Chumanevich *et al.*, 2004 ▶), Nt-FDH from *Homo sapiens* (*Hs*_Nt-FDH; Kursula *et al.*, 2006 ▶), FMT from *E. coli* (*Ec*_FMT; Schmitt *et al.*, 1996 ▶), ArnA-TF from *E. coli* (*Ec*_ArnA-TF; Gatzeva-Topalova *et al.*, 2005 ▶) and GART from *Symbiobacterium toebii* (*St*_GART; Yoshizawa *et al.*, 2011 ▶). The completely conserved residues are shown on a red background. The residues of zNt-FDH involved in ligand binding *via* hydrogen bonds, according to the protein structures of the 10-­FDDF and THF complexes, are marked by stars and squares, respectively. The green box indicates the highly conserved region with the H*x*SLLP*xxx*G sequence motif. The sequence alignment was performed with *STRAP* and *ClustalW*. The secondary-structure elements of *Zf*_Nt-FDH were calculated with *ESPript* (http://espript.ibcp.fr) and are presented at the top of the sequences.

We have determined the apo-form structure of zNt-FDH_WT at a resolution of 1.75 Å. The overall structure of zNt-FDH is divisible into N- and C-sub­domains, which are also found in Nt-FDH from rat and human as well as FMT (Chumanevich *et al.*, 2004 ▶; Kursula *et al.*, 2006 ▶; Schmitt *et al.*, 1996 ▶). The N-subdomain, a folate-binding domain, comprises a six-stranded β-sheet (parallel β1–β4 and antiparallel β5–β6) and six α-helices (α1–α6) and forms a Rossmann fold (Fig. 1 ▶ *a*). A structural comparison of all 10-formyltetrahydrofolate-utilizing enzymes (Fig. 2 ▶) showed that the r.m.s. deviation (r.m.s.d.) of this six-stranded sheet in the folate-binding domain is 0.31–0.62 Å. The C-subdomain has mostly β-strands in the structure and contains an open barrel-like domain of two β-sheets: one five-stranded sheet (β9–β13) and one two-stranded sheet (β7–β8). The superposition of Nt-FDH structures from zebrafish (this work), rat (r.m.s.d.s of 0.51 and 0.49 Å for the N- and C-subdomains, respectively; Chumanevich *et al.*, 2004 ▶) and human (0.70 and 0.46 Å; Kursula *et al.*, 2006 ▶) and the other folate-utilizing proteins FMT (1.20 and 2.14 Å), ArnA (PmrI) transformylase (ArnA-TF; 2.09 and 2.28 Å) and GART (3.69 Å, no C-subdomain) (Schmitt *et al.*, 1996 ▶; Gatzeva-Topalova *et al.*, 2005 ▶; Yoshizawa *et al.*, 2011 ▶) reveals similarity and deviations, with r.m.s.d.s ranging from 0.5 to 3.7 Å for N-subdomains and from 0.46 to 2.28 Å for C-subdomains, despite the low sequence identity of zNt-FDH to FMT, ArnA-TF and GART (∼27%). Three loops containing residues 86–90, 135–143 and 200–203 near the binding cavity, located in the Rossmann-fold subdomain, accommodate substrate or product binding as well as the catalytic triad in the active site (Figs. 1 ▶ *a* and 1 ▶ *b*). All superimposed structures of these enzymes (Fig. 2 ▶) showed an r.m.s.d. of 0.33 Å or less for these three loops.

The structures of Nt-FDH from rat (Chumanevich *et al.*, 2004 ▶) and human (Kursula *et al.*, 2006 ▶) have previously been documented. The structure complexed with THF has only been reported for the human enzyme (PDB entry 2cfi; Kursula *et al.*, 2006 ▶), in which the electron density of THF in the active site showed only the pteridine moiety (6-formyltetrahydro­pterin). To obtain further information about the catalytic mechanism, we solved various structures of Nt-FDH complexes with the substrate and products in their complete forms, which are described in detail in the following sections.

### 3.2. Sequence alignment and catalytic residues   

A comparison of the N-terminal hydrolase domain of FDH from zebrafish (zNt-FDH) with other 10-formyltetrahydro­folate-binding enzymes reveals sequence identities between zNt-FDH and Nt-FDH from *Rattus norvegicus* (73%; Chumanevich *et al.*, 2004 ▶), Nt-FDH from *Homo sapiens* (73%; Kursula *et al.*, 2006 ▶), FMT from *E. coli* (27%; Schmitt *et al.*, 1996 ▶), ArnA-TF from *E. coli* (20%; Gatzeva-Topalova *et al.*, 2005 ▶) and GART from *Symbiobacterium toebii* (20%; Yoshizawa *et al.*, 2011 ▶) (Fig. 2 ▶). Sequence alignment of these enzymes along with their structural conservation suggests that the sequence motif H*x*SLLP*xxx*G is conserved in the Rossmann fold (Gatzeva-Topalova *et al.*, 2005 ▶). These six enzymes contain the conserved ^106^H*x*SLLP*xxx*G^115^ motif (numbers refer to the sequence of zNt-FDH) and are annotated as formyltransferases (Gatzeva-Topalova *et al.*, 2005 ▶). Situated before this specific motif, the residue Ile104 (residue numbers refer to zNt-FDH in the following) in zNt-FDH, human Nt-FDH and rat Nt-FDH, together with the corresponding Asn residue in FMT, ArnA-TF and GART, might play an important role in the hydrolase and transferase catalytic mechanisms in the homologous enzymes. This Asn is a crucial residue for catalysis in these 10-formyltetra­hydrofolate-utilizing enzymes (FMT, ArnA-TF and GART), and it has been proposed that it stabilizes the formyl group of 10-formyltetrahydrofolate with the protonated imidazolium of His106, enabling their respective substrates to be formylated on nucleophilic attack (Shim & Benkovic, 1999 ▶; Newton & Mangroo, 1999 ▶; Reuland *et al.*, 2006 ▶). Likewise, mutating Ile104 to alanine and asparagine decreased the hydrolase activity of the full-length FDH to ∼50 and <10%, respectively (Reuland *et al.*, 2006 ▶). The SLLP polypeptide motif adopts a type VIa turn, in which Pro111 is involved in a *cis*-peptide bond (Gatzeva-Topalova *et al.*, 2005 ▶). Ser108 does not interact directly with the substrate or product according to our structures, but it is located near the catalytic residue His106 and has been proposed to help the electrons to rearrange in the hydrolytic reaction (Reuland *et al.*, 2006 ▶). The replacement of Ser108 by alanine caused the hydrolytic activity to decrease to below 15% (Reuland *et al.*, 2006 ▶). It has been proposed that Leu109 and Leu110 supply the hydrophobic environment for ligand binding (Reuland *et al.*, 2006 ▶). Two catalytically important and highly conserved residues, Asp142 and His106, have been identified in the hydrolytic site of FDH. These two residues are located in the folate-binding cavity of the superimposed structures of zNt-FDH and other 10-formyl­tetrahydrofolate-binding enzymes, implying a similarity in catalytic mechanism. His106, Ser108 and Asp142 in the active site might act as a kind of catalytic triad.

### 3.3. 10-FDDF and formate binding in the active site   

**Figure 3:**
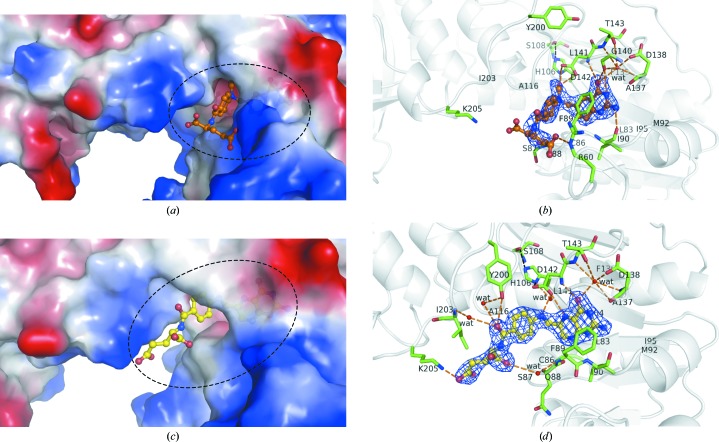
The substrate and products in the active-site pocket of zNt-FDH. (*a*) Electrostatic surface potential (blue, positive charge; red, negative charge) of the 10-FDDF complex of Nt-FDH. The bound 10-FDDF (orange) is shown in ball-and-stick representation. (*b*) Interactions between zNt-FDH and 10-­FDDF. Hydrogen bonds between residues and between residues and 10-FDDF are presented as black and orange dotted lines, respectively. Two residues (His106 and Asp142) are reported to participate in the catalytic mechanism of both the dehydrogenase and the hydrolase reaction in FDH (Krupenko & Wagner, 1999 ▶; Krupenko *et al.*, 2001 ▶). The ND1 atom of His106 and the OD1 and OD2 atoms of Asp142 interact directly with the OA1 atom of the N10 formyl group. (*c*) Electrostatic surface potential of the zNt-FDH–THF complex. The bound THF (yellow) is shown in ball-and-stick representation. (*d*) Interactions between zNt-FDH and THF. Hydrogen bonds between residues and between residues and THF are presented as orange dotted lines. The THF-bound form of zNt-FDH demonstrate that the conformational change of Tyr200 stabilizes the O atom of pABA, such that the –­OH group of Tyr200 can directly interact through hydrogen bonding. All of the 2*F*_o_ − *F*_c_ electron-density maps are contoured at 1.0σ.

To obtain detailed information on the substrate-binding site of zNt-FDH, we have determined structures of the protein complexed with 10-FDDF, a 10-FTHF analogue with higher stability. Previous studies have shown that 10-FDDF bound to FDH and was catalysed readily (Krupenko *et al.*, 1995 ▶; Fu *et al.*, 1999 ▶). The 10-FDDF is complete and kinked in this subdomain with the Rossmann fold of zNt-FDH (Fig. 3 ▶ *a*). The bicyclic pteridine ring is well ordered (*B* factor of ∼30 Å^2^), whereas the structure towards the pABA (∼50 Å^2^) and glutamate group (∼71 Å^2^) is relatively unstable. The detailed interactions between 10-FDDF and zNt-FDH are shown in Fig. 3 ▶(*b*). The pteridine moiety of 10-FDDF interacts with the main-chain carbonyl groups of Ile90, Asp138 and Gly140 and the amide group of Asp142 through direct hydrogen bonds and with the main-chain amide or carbonyl of Asp138, Gly140 and Thr143, the OD2 atom of Asp138 and the OG1 atom of Thr143 through water-mediated hydrogen bonds (Table 2 ▶). Leu83, Phe89, Met92, Ile95, Ile104, Phe135, Ala137 and Leu141 stabilize the pABA moiety of the 10-FDDF through hydrophobic interactions. His106 and Asp142 have been reported to participate in the catalytic mechanism of both the dehydrogenase and hydrolase reactions in FDH (Krupenko & Wagner, 1999 ▶; Krupenko *et al.*, 2001 ▶). The OD1 and OD2 atoms of Asp142 and the ND1 atom of His106 interact directly with the OA1 atom of the N10 formyl group, increasing its nucleophilic character and releasing the N10 formyl group (–­CHO) from the substrate during the hydrolase reaction (Table 2 ▶). The main-chain carbonyl group of Ser87 and the side chain of Arg60 interact directly with the glutamate group through hydrogen bonding (Table 2 ▶). The structure from N10 to the glutamate moiety is also stabilized by Cys86, Gln88, Gly115, Ala116 and Ile203 through hydrophobic interactions.

In the active site, the formyl group of the substrate is converted into the product formate during catalysis. In the structure of the formate complex, the formate molecule interacts with His106 and Asp142. The O1 atom of formate is directly hydrogen-bonded to OD2 of Asp142 (within 2.73 Å). The O1 atom of the formate interacts with the ND1 atom of His106 and the OD1 atom of Asp142 at distances of 3.46 and 3.19 Å, respectively.

### 3.4. THF binding in the active site   

The structure of each monomer in the THF-bound form of zNt-FDH is nearly identical to the apo form, with an r.m.s.d. of 0.65 Å for 308 C^α^ atoms; the central hole has a solvent-accessible pocket of similar size in the structures of both apo Nt-FDH and the THF complex. In the structure of the zNt-FDH–THF complex, we found that THF adopts a kinked conformation in the active site (Figs. 3 ▶ *c* and 3 ▶ *d*). The structure of THF is complete, in contast to that of the human structure, in which only 6-formyltetrahydropterin was observed (Kursula *et al.*, 2006 ▶), providing complete information on the inter­action between THF and its surrounding residues.

The pteridine ring of THF is buried in the central cavity and makes a stacking interaction with the side chain of Phe89. The pteridine moiety of THF is recognized by the main-chain carbonyl groups of Gln88, Ile90, Asp138 and Gly140 and the amide group and OD1 atom of Asp142 through direct hydrogen bonds and by the main-chain amide or carbonyl of Asp138, Gly140, Thr143 and Gly144, the OD1 atom of Asp138 and the OG1 atom of Thr143 through water-mediated hydrogen bonds (Table 2 ▶). In this environment, some residues, including Leu83, Cys86, Met92, Ile95, Ile104, Phe135, Ala137 and Leu141, also provide hydrophobic interactions to stabilize the pteridine group. The hydroxyl O atom of Tyr200 exhibits direct and water-mediated interactions with the O atom of the pABA moiety within each monomer of the dimer, whereas the main-chain atoms of Ala116, Glu201 and Ile203 interact indirectly through water molecules with the O atom of the pABA within a hydrogen-bond distance (Table 2 ▶).

Structural investigations of apo and THF-bound zNt-FDH demonstrate that the conformation of Tyr200 is altered, in which the –OH group of Tyr200 can make a direct or water-mediated interaction through a hydrogen bond to stabilize the O atom of pABA. Cys86 and Ser87 of the β5–α4 loop and Ala116 of the β6–α5 loop interact with the pABA group through hydrophobic interactions. The main-chain amide groups of Phe89 and Ile203 mediate the water molecules through hydrogen bonds to interact with the O1 and O2 atoms of the THF glutamate, respectively (Table 2 ▶). The OE1 atom of the glutamyl group of THF also makes a salt bridge with the side chain of Lys205.

### 3.5. Comparison of THF and 10-FDDF in the active site of zNt-FDH   

**Figure 4:**
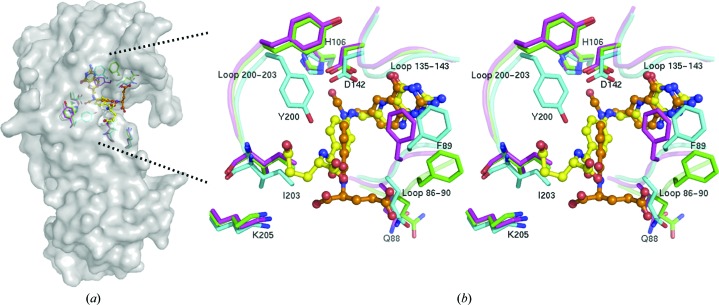
Superimposition of residue side chains in the active site for apo zNt-FDH and the zNt-FDH–THF and zNt-FDH–10-FDDF complexes. (*a*) zNt-FDH surface structure with 10-FDDF and THF bound in the active site. (*b*) A stereoview of conformational changes in the active site between the apo form and the 10-­FDDF and THF complexes. 10-FDDF (orange) and THF (yellow) are shown in ball-and-stick representation. Residues of zNt-FDH (green), the 10-­FDDF complex (magenta) and the THF complex (cyan) are shown as stick representations. The three loops 86–90, 135–143 and 200–203 are closed to the binding cavity with the product THF and substrate 10-FDDF bound. In the structure of THF-bound zNt-FDH, the conformation of the side chain of Tyr200 rotates by 90° to facilitate product binding.

Structures of the substrate (10-FDDF) and product (THF) complexes were obtained by the crystal-soaking method with crystals of apo-form zNt-FDH_WT and zNt-FDH_Y200A. Adjacent to the active site, there is a positively charged patch which may be involved in the binding of the polyglutamate group. Notably, in a comparison of the structures of zNt-FDH_WT, the side-chain conformations of Tyr200 in the apo form and the complex with 10-FDDF are similar, whereas Tyr200 exhibits a distinct conformation in the structure of the THF complex (Fig. 4 ▶). In the structure of THF-bound zNt-FDH, the aromatic ring of Tyr200 is observed to rotate by 90° to make space for product binding. The position of the side chain of Tyr200 in the THF product complex differs from that in the structures of the apo form and the complex with the substrate 10-FDDF, leading to speculation that Tyr200 might be related to product release.

The three loops containing residues 86–90, 135–143 and 200–203 are positioned near the binding cavity upon binding of THF and 10-FDDF. The side chain of Phe89 is rotated and its position is shifted to stabilize the pteridine group of THF and 10-FDDF in a comparison of the structures of the apo form and the complexes. In the structures of the complexes, the loop ^86^CSQFI^90^ moves close to the substrate and product with a maximum distance of ∼2.8 Å within the active site and the side chain of Phe89 is altered to form a π–π interaction with the pteridine moiety (Fig. 4 ▶).

The main feature of the differences between the artificial substrate 10-FDDF and the natural product THF is that the N5 and N8 atoms of the bicyclic ring in THF are replaced by C atoms in 10-FDDF, which provide the formyl group at the N10 position. The structural results indicate that the pteridine moieties of 10-FDDF and THF could be well aligned (Fig. 4 ▶). The C6—C9—N10 bond angles in 10-FDDF and THF are 114.3 and 111.0°, respectively. The most evident positional differences between 10-FDDF and THF are located mainly within the pABA and glutamate moieties. The difference in the pABA ring angle between 10-FDDF and THF is ∼9.2°.

The glutamate is shifted by ∼4.7 Å from a position near the loop of residues 86–90 to a position near the loop of residues 200–203 based on measurements of the CA atom of the glutamate in 10-FDDF and THF. His106 and Asp142 interact with the formyl group of 10-FDDF through hydrogen bonds, whereas the interactions of His106 and Asp142 with THF are mediated by one water molecule within a hydrogen-bond distance attempting to mimic and replace the O atom of the formyl group in the active site (Fig. 4 ▶). In the case of THF, Asp142 also interacts with the N5 atom of the product through a direct hydrogen bond, but this N atom is replaced by a C atom in 10-FDDF. Tyr200 exhibits direct and water-mediated interactions with the O atom in the pABA moiety of THF, but there is no interaction between Tyr200 and 10-FDDF (Fig. 3 ▶). The main-chain carbonyl group of Ser87 and the side chain of Arg60 interact directly with the glutamate group of 10-FDDF through hydrogen bonding. In contrast, the main-chain amide group of Phe89 interacts with the O1 atoms of the THF glutamate through hydrogen bonds mediated by water molecules. A comparison of the catalytic triad in the active site of the apo and the complexes (with 10-FDDF and THF) shows that the position of the catalytic residue His106 is essentially unchanged but that Ser108 and Asp142 are shifted toward the product by ∼0.9 and 0.8 Å, respectively, in the structure of the THF complex (Fig. 4 ▶).

### 3.6. The additional potential THF binding site of zNt-FDH   

**Figure 5:**
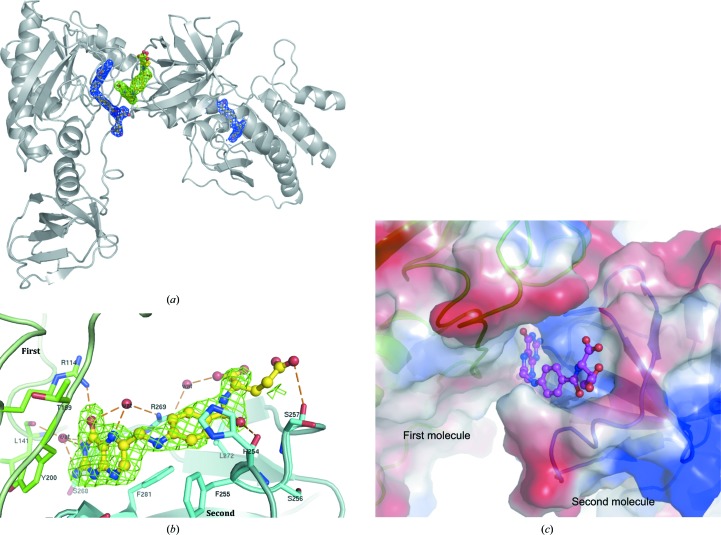
Additional potential THF binding site in zNt-FDH. (*a*) An additional THF binding site with clear electron density (green mesh) for THF is observed in the cavity between two zNt-FDH molecules. The THF molecules in the active sites are shown with electron density (blue mesh). The 2*F*_o_ − *F*_c_ electron-density maps are contoured at 1.0σ. (*b*) The omitted *F*_o_ − *F*_c_ electron-density map of THF (yellow ball-and-stick representation) is contoured at 2.0σ. This additional THF is stabilized by the interaction between two zNt-FDH molecules. One molecule is shown in cyan and the other in green. Hydrogen bonds are presented as orange dotted lines. (*c*) The electrostatic surface (blue, positive charge; red, negative charge) of the additional THF binding site with bound THF (magenta ball-and-stick representation). Two zNt-FDH molecules are shown in green and cyan, respectively, in ribbon representation.

An extra electron density representing THF was clearly observed in the groove between two zNt-FDH monomers, in addition to that in the catalytic site, when crystals of apo zNt-FDH_WT were soaked with THF (Figs. 5 ▶ *a* and 5 ▶ *b*). The space group of this THF complex was transformed from the *P*2_1_2_1_2 space group of the apo form, containing one zNt-FDH molecule in the asymmetric unit, to *P*2_1_2_1_2_1_ with two molecules in the asymmetric unit (Table 2 ▶). The average *B* factor (52 Å^2^) of the THF in the cleft near the interface between two zNt-FDH molecules is greater than that of the THF in the active site (32 Å^2^), suggesting relatively weak binding of the second THF molecule. This secondary binding of THF is probably a crystallization artifact or acts as a potential storage binding of folate in cells, which might play an important role in regulating the amounts of THF in the one-carbon metabolism (Kim *et al.*, 1996 ▶), as discussed further in §[Sec sec4.5]4.5.

The additional THF is stabilized by interactions between two zNt-FDH molecules (Fig. 5 ▶ *b*). The pteridine moiety interacts with the NH2 atom of Arg114 (first molecule) and the main-chain carbonyl group of Gly282 (second molecule) through a direct hydrogen bond as well as with the NH2 atom of Arg269 (second molecule) and the main-chain carbonyl groups of Leu141 (first molecule) and Ser268 (second molecule) through water-mediated hydrogen bonds (Table 2 ▶). Tyr200, Thr199 (first molecule) and Phe281 (second molecule) provide hydrophobic interactions to stabilize the pteridine part in this additional THF-binding pocket. His254, Phe255, Leu272 and Phe281 from the second protein molecule interact mainly with the pABA moiety of THF through hydrophobic forces. Finally, the glutamate group of THF is directly hydrogen-bonded to the main-chain amide group of Phe255, the O atom of Ser257 and the NH1 atom of Arg269 from the second molecule (Table 2 ▶). A comparison of the structural conformations with temperature *B* factors for the THF molecules bound to the active site and the secondary THF binding site reveals that the active-site THF is more stable than the additionally bound THF; the glutamate (*B* factors of 48.5 and 80.7 Å^2^ for the active and additional binding sites) and pABA parts (25.6 and 54.2 Å^2^) are more dynamic than the pteridine (21.3 and 28.6 Å^2^) moieties in both the active and secondary THF binding sites.

The electrostatic surface near the second THF binding site clearly shows that the THF binds to the pocket formed by two molecules (Fig. 5 ▶ *c*). The pteridine moiety and the pABA part interact with several hydrophobic and hydrophilic residues, and are buried in the deep pocket of the active site (Fig. 3 ▶ *d*) or the additional THF binding site (Fig. 5 ▶ *b*). As mentioned previously, the catalytic site is more positively charged, providing an environment for tight binding of the THF glutamate moiety (Fig. 3 ▶ *c*). The second binding site, on the other hand, is more negatively charged to allow weak binding of the glutamate moiety, which is in agreement with the larger *B* factor for THF observed at this site. Unexpectedly, despite the structural similarity between the folate and the folate analogue (Supplementary Fig. S1), 10-FDDF was not observed in the second binding site when 10-FDDF was used in place of THF for crystal soaking, suggesting THF binding specificity for the second binding site (Supplementary Fig. S1). This additional THF binding site also provides a possible explanation and mechanism for the product inhibition of FDH, which is further discussed below.

### 3.7. Functional analysis of zNt-FDH   

Based on the obtained structures of zNt-FDH and its complexes, we constructed and analysed several zNt-FDH mutants encompassing specific site-directed mutations of the residues surrounding the substrate-binding and product-binding pockets. The *K*_m_ and the *k*_cat_ for the hydrolase activity of wild-type zNt-FDH towards 10-FTHF are 12.7 µ*M* and 13.0 min^−1^, respectively (Chang *et al.*, 2010 ▶). The activity assay shows that the hydrolase activities of the F89A, R114A and Y200A zNt-FDH mutants decreased significantly in comparison to that of zNt-FDH_WT (Table 3 ▶). Structural analysis of the zNt-FDH complexes with 10-FDDF and THF reveals that these residues form hydrophobic interactions or hydrogen bonds with the substrate or product, suggesting that they are important for stabilizing the ligand binding for catalysis. Phe89, Arg114 and Tyr200 are highly conserved in FDH from all species. In the complex structures with THF or 10-FDDF, Phe89 appears to interact with the pteridine moiety through a stacking force. The F89A mutation decreased the enzymatic activity by about 85%, implying that a hydrophobic residue might be essential in this region for efficient catalysis. The NE atom of Arg114 interacts by hydrogen bonding to the O atom of Asp142, which is a crucial catalytic residue in the active site. Arg114 might help to stabilize Asp142 in the active site for catalysis. When Arg114 is mutated to alanine, the activity assays show that the enzymatic activity is greatly decreased. Although Lys205 directly interacts with the OE1 atom of the glutamyl group of THF, the disordered glutamate moiety of THF might reflect its flexibility, allowing proper positioning of the reactive groups for a direct nucleophilic attack on the N10 atom of the substrate and release of the formyl group. Lys205, with weak interactions with the glutamyl group, does not seem to be involved in catalysis because the enzymatic activity is not notably affected when this residue is mutated to alanine.

### 3.8. Potential protein dimerization induced by product binding   

The association between zNt-FDH and THF was measured by dynamic light scattering (DLS), which provides the hydrodynamic radius (*R*_h_), molecular mass (MW) and mass distribution of polymerization for the zNt-FDH–THF complex in solution. The ligand-free form of zNt-FDH shows a mean *R*_h_ of 2.7 nm, which is in satisfactory agreement with the estimated value of 3.0 nm deduced from the structure of zNt-FDH using *HYDROPRO* (Ortega *et al.*, 2011 ▶). When the product THF (at 1 µ*M* to 1 m*M*) is added to zNt-FDH (2 mg ml^−1^), the predominant size of the Nt-FDH–THF complex in solution increased to 3.9 ± 0.1 nm (Table 4 ▶), which is in good correspondence to the value of 4.0 nm calculated for the dimer with *HYDROPRO*. When the zNt-FDH and THF molar ratios are varied from 1:0 to 1:20, a transition between monomers and dimers is observed at molar ratios between 1:1 and 1:2 (Supplementary Figs. S2 and S3), with the *R*_h_ of zNt-FDH–THF showing sizes of both the monomer and the dimer. The sample exhibits severe light scattering when the concentration of THF exceeds 1 m*M* (molar ratio of 1:20). At a high concentration (100 µ*M* to 1 m*M*) of THF, the average *R*_h_ of the nanoparticles increases further by 1.1–1.3 nm from that of the THF-free zNt-FDH. This enhancement of the particle sizes in solution is presumably owing to the formation of protein complexes, suggesting that the close association between two molecules to induce partial dimerization of zNt-FDH is affected by THF. Similar experiments were conducted with the substrate 10-FDDF, but no dimerization phenomenon was observed, indicating that only the product can induce protein dimerization.

## 4. Discussion   

### 4.1. The effect of β-mercaptoethanol on zNt-FDH   

The crystallization and soaking conditions for the determination of our complex structures with 10-FDDF and THF (20 m*M* Tris–HCl buffer pH 7.5, 300 m*M* NaCl) differ from those previously used for Nt-FDH from rat (20 m*M* Tris–HCl buffer pH 7.5, 10 m*M* β-mercapto­ethanol) and human [20 m*M* HEPES pH 7.5, 300 m*M* NaCl, 10% glycerol, 2 m*M* tris(2-carboxyethyl)phosphine (TCEP)]. β-Mercaptoethanol and 6-formyltetrahydropterin (an incomplete folate) were previously found to occupy the active sites of rat and human Nt-FDH, respectively (Kursula *et al.*, 2006 ▶; Chumanevich *et al.*, 2004 ▶). In the current work, we have obtained two new complex structures with 10-FDDF and THF representing different states before and after catalysis. Without the addition of β-mercaptoethanol, the structure of the 10-FDDF complex is intended to represent the substrate-bound form before the hydrolase reaction, whereas that of the THF complex describes the product-bound form after the hydrolase reaction.

**Figure 6:**
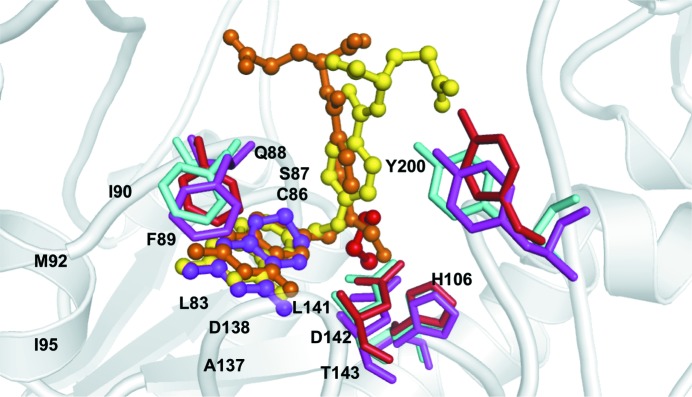
Structural comparison of the zNt-FDH–10-FDDF/THF complex with the human Nt-FDH–6-formyltetrahydropterin and rat Nt-FDH–β-mercapto­ethanol complexes. 10-FDDF (orange), THF (yellow), 6-formyltetra­hydropterin (magenta) and β-mercaptoethanol (red) are shown in ball-and-stick representation. Residues in the zNt-FDH–THF (cyan), human Nt-FDH–6-formyltetrahydroptein (magenta) and rat Nt-FDH–β-­mercaptoethanol (red) structures are shown in stick representation.

In attempts to soak 10-FDDF or THF into the apo zNt-FDH crystals under conditions with β-mercaptoethanol for structure analysis, we found no clear extra electron density for 10-FDDF or THF in the active site (data not shown). Instead, a β-mercaptoethanol molecule is observed at a position similar to that observed in the active site of the rat Nt-FDH structure. β-Mercaptoethanol is considered to be a cofactor in enzyme catalysis, but it might affect crystallization in the states before and after catalysis of zNt-FDH. The conditions of our protein solution for crystallization do not contain any reducing agent (β-mercaptoethanol or TCEP), and in this manner we successfully obtained the complete structures with 10-FDDF and THF in the active site. Fig. 6 ▶ shows a comparison of the active sites of zNt-FDH complexes with 10-FDDF and THF, the human Nt-FDH complex with 6-formyltetrahydro­pterin and the rat Nt-FDH complex with β-mercaptoethanol. 6-Formyltetrahydropterin is located at the same position as the pteridine moiety of 10-FDDF and THF, whereas β-mercaptoethanol is present at a position similar to that of the N10 formyl group, but the role of β-mercapto­ethanol in the catalytic mechanism of FDH remains unclear.

Previous studies showed that the NADP^+^-independent hydrolysis of 10-FTHF catalysed by either full-length FDH or Nt-FDH *in vitro* requires the presence of β-mercaptoethanol at millimolar levels (Schirch *et al.*, 1994 ▶), which, however, is unlikely to occur in living cells. A hypothesis has been proposed in which β-mercaptoethanol is required for replacing and releasing the product from the catalytic site because of the structural similarity between β-mercaptoethanol and the product formate (Chumanevich *et al.*, 2004 ▶). It is possible that the absence of β-mercaptoethanol in our crystallization and soaking conditions resulted in the substrate and product being stably retained in the active site in our crystal structures.

### 4.2. Substrate/product and the active site of zNt-FDH   

The structures of the complexes with 10-FDDF and THF show that the pABA and the glutamate group exhibit larger *B* factors than the pteridine moieties of both 10-FDDF and THF, indicating that the pABA and the glutamate group are more dynamic than the pteridine moiety. The pteridine group is stably buried in the active site. The structural flexibility at the end of the ligands might facilitate nucleophilic attack on the formyl group and transfer of the formyl group to the intermediate domain of FDH. This domain requires a 4′-PP prosthetic group covalently bound to Ser354 in rat Nt-FDH (Ser355 in zNt-FDH) and is proposed to act as a swinging arm to transfer the formyl group to the C-terminal dehydro­genase domain coupling NADP^+^ for the conversion of 10-FTHF to THF and CO_2_ (Krupenko, 2009 ▶; Strickland *et al.*, 2010 ▶). An inspection of the structures of the ligand-free apo zNt-FDH and its respective complexes with 10-FDDF and THF reveals that the catalytic residue His106 remains in a similar position, whereas the positions of Ser108 and Asp142 are shifted towards the formyl group by ∼0.9 and 0.8 Å, respectively, in the active site as observed in the structure of the THF complex. The electrostatic potential surface of zNt-FDH shows a net positive charge outside the folate-binding site under neutral pH as previously described for a possible cationic binding locus to stabilize the extended negatively charged polyglutamate tail of H_4_PteGlu_*n*_ that protrudes from the folate-binding site (Fig. 3 ▶; Fu *et al.*, 2003 ▶).

**Figure 7:**
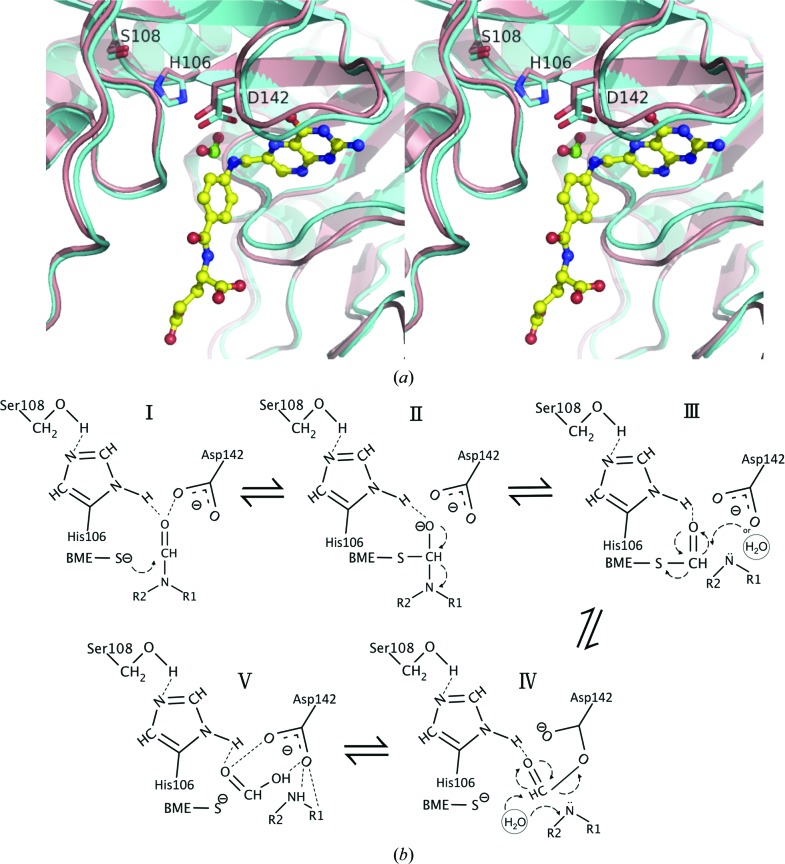
The proposed mechanism of zNt-FDH based on superimposition of formate and THF in the active site between the structures of the zNt-FDH–formate and the zNt-FDH–THF complexes. (*a*) A comparison of the structures of the formate complex (green C atoms) and THF complex (yellow C atoms) forms. Both formate and THF molecules are shown in ball-and-stick representation; the residues His106, Ser108 and Asp142 of the formate complex (salmon) and the THF complex (cyan) are shown in stick representation. (*b*) The S atom of β-mercaptoethanol (BME) attacks the substrate (10-FTHF) to form an intermediate state and release the product (THF). Asp142 activates the water molecule or directly attacks the C atom of the formyl group. BME and the formyl group bind to Asp142; thus, breakage of the sulfur–carbon bond underlies the biochemical process. The water molecule attacks the formyl group to form the formate during the hydrolase reaction. After hydrolysis, the products (formate and THF) can be stabilized by the catalytic residues (His106 and Asp142) in the active site.

In the active site, Ser108 is hydrogen-bonded to His106, which is further hydrogen-bonded to Asp142. The ligand–protein interactions in the respective structures of the zNt-FDH complexes with 10-FDDF (Figs. 3 ▶ *a* and 3 ▶ *b*), THF (Figs. 3 ▶ *c* and 3 ▶ *d*) and formate could provide us with some structural information on the catalytic mechanism through a comparison of the active sites of various complex structures (Fig. 7 ▶ *a*). Fig. 7 ▶(*b*) presents our proposed mechanism in five different states. His106 and Asp142 help to orient the carbonyl O atom of the substrate (Fig. 7 ▶ *b*, step I). β-Mercaptoethanol includes the thiol group that first attacks the substrate (10-FTHF) to form an intermediate state and then releases the THF (Fig. 7 ▶ *b*, step II). There are two possible pathways for catalyzing the formyl group. One was proposed in the previous studies on the rat and human enzymes (Chumanevich *et al.*, 2004 ▶; Kursula *et al.*, 2006 ▶), in which Asp142 first polarizes a water molecule to initiate the nucleophilic attack on the carbonyl C atom of the substrate. Our 10-FDDF complex structure, without an observed activated water in the active site, suggests another possible pathway which invokes the carboxylate of Asp142 as a nucleophile to directly attack the C atom of the formyl group. Since we do not see the water molecule in question in the 10-FDDF complex, we could not exclude the possibility of the first mechanism. The chemical structures of the substrates and the product indicate that the pteridine moiety of 10-FDDF is more hydrophobic than 10-FTHF and THF (Supplementary Fig. S1). Thus, there is a possibility that the water molecule is associated with the 10-FTHF. In step III, cleavage of the activated formyl group at the S atom splits the substrate into β-mercaptoethanol and the transition-state formyl group that is bound to Asp142. A water molecule then attacks the formyl group to give the formate in the hydrolase reaction (Fig. 7 ▶ *b*, step IV). After hydrolysis, the products (formate and THF) could be stabilized by the catalytic residues (His106 and Asp142) in the active site (Fig. 7 ▶ *b*, step V). For the full-length FDH, the prosthetic group of 4′-PP might play the same role as β-mercaptoethanol by providing the thio group in the hydrolase reaction.

### 4.3. The potential role of Tyr200 in the catalytic mechanism   

Our results suggest that Tyr200 might play a crucial role in the release of products. The side chain of Tyr200 exhibits the same conformation in the structures of zNt-FDH with and without the substrate 10-FDDF before hydrolysis occurs, but this aromatic ring of Tyr200 is flipped about 90° from the surface inwards into the active site upon product binding, as observed in the structure of the product complex. We observed that Tyr200 does not directly interact with the bound substrate in the zNt-FDH_WT–10-FDDF complex and that 10-FDDF could still bind in the active site of zNt-FDH_Y200A, implying that Tyr200 does not affect the initial substrate-binding ability.

The conformational differences in the active site between the 10-FDDF and THF complex structures imply that a specific coordinated motion is associated with the catalysis reaction from the substrate to the product. This observation suggests that Tyr200 might participate in product release. As expected, the Y200A mutation decreased the enzymatic activity by ∼62%, preventing us from examining the kinetic properties of the mutant FDH (Table 3 ▶). We co-crystallized or soaked crystals of the mutant (Y200A) with the product THF, but we observed no clear extra electron density in the active sites of the structures. The buried surface area of the THF complex (∼310 Å^2^) is less than that of the 10-FDDF complex (∼360 Å^2^) by 50 Å^2^. This decreased surface area buried in the structure of zNt-FDH is a reflection of conformational alteration of the side chain of Tyr200. This Tyr residue is highly conserved in zNt-FDH, rat Nt-FDH, human Nt-FDH and *E. coli* FMT, as shown in Fig. 2 ▶. During hydrolysis of the formyl group, the positional shifts of the pABA and the glutamic acid moiety, together with the conformational alteration of Tyr200, might facilitate the departure of THF from the catalytic binding site (Fig. 4 ▶).

### 4.4. Roles of catalytic residues   

On the basis of our structures of the protein complexes with the substrate 10-FDDF and the product THF, we selected several residues located in the active or additional THF binding sites to examine their functional roles in catalysis. We mutated Phe89, Arg114, Tyr200 and Lys205 to Ala in zNt-FDH and assayed the activities of the corresponding mutants (Table 3 ▶). The F89A, R114A and Y200A mutations decrease the enzymatic activity, whereas the K205A mutation does not significantly affect the activity. The glutamate group of the ligand is flexible towards the surface as shown in the complex structures, but Phe89 could interact with the pteridine moiety of the substrate or product, making the pteridine moiety more stable in the active site for the hydrolase reaction. Arg114 is located on the surface of the protein; it provides hydrogen bonding to the bound THF at the secondary or ancillary THF binding site and interacts with the catalytic residue Asp142 for catalysis. The exact role of this Arg residue is not clearly understood at present. Finally, from our structures, we have identified a new key residue Tyr200 that might be involved in product release, product inhibition and additional THF binding. The structural information, together with the mutation and activity assays, provide new insights towards understanding the role of FDH in the folate-mediated one-carbon metabolism.

### 4.5. The additional potential THF binding site of zNt-FDH   

**Figure 8:**
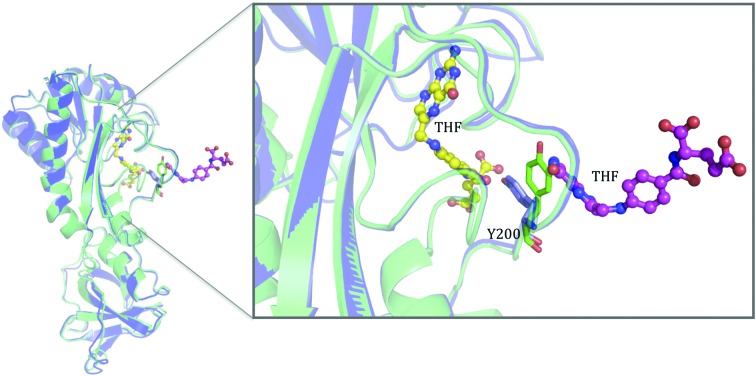
Potential product inhibition of Nt-FDH. Comparison of the overall structure of the apo (green) and THF-complexed (blue) forms: the THF molecules are shown in ball-and-stick representation and the Tyr200 residue of the apo form and the THF complex form are shown in stick representation. In a close-up view of the active site, the additional bound THF occupies a position that interferes with Tyr200 if the orientation of the aromatic group is not altered from that in the native structure or that of the substrate (10-FDDF) complex.

Based on our structural analysis and mutagenesis studies, we propose another possible mechanism for the product inhibition of FDH which involves the binding of THF to the potential second binding site. Zebrafish FDH exhibits product inhibition, in which a rapid decrease in the catalytic activity was observed upon accumulation of THF in the activity assay (Chang *et al.*, 2010 ▶). In our proposed model, the additional THF bound to the second site would structurally interrupt the switch in orientation of the aromatic side chain of Tyr200, which is the crucial step for product release and substrate binding, hence preventing release of the product from the active site. This is because binding of THF to the second site would leave insufficient space for the aromatic side chain of Tyr200 to flip and return to its initial position (apo form or substrate-binding structure) after the catalytic reaction. This structural limitation thus retains Tyr200 in the state found in the complex with product, hindering the entrance of new substrate into the active site and slowing down the catalytic reaction (Fig. 8 ▶). This proposition is partly supported by the previous reports that adding SHMT to the reaction mixture of FDH removed product inhibition (Chang *et al.*, 2010 ▶). FDH purified from rabbit liver contains tightly bound THF polyglutamate (*K*_d_ = 15 n*M*) and exhibits product inhibition. It has been postulated that the product inhibition might be owing to stronger binding of the product to the enzyme compared with the substrate (Kim *et al.*, 1996 ▶). Analysis of the THF binding stoichiometry of rabbit FDH also revealed the existence of an additional THF binding site besides the substrate catalytic site in the N-terminal domain of FDH (Fu *et al.*, 1999 ▶). The structures of the product complexes presented in the current study provide additional insights into the possible underlying mechanisms of production inhibition.

The ability of FDH to bind THF and act as a storage reservoir of THF is crucial for normal physiological function and the pathological mechanism. In addition to serving as a one-carbon carrier, THF is also a potent antioxidant both *in vitro* and *in vivo* (Kao *et al.*, 2014 ▶; Rezk *et al.*, 2003 ▶). Overexpressing FDH increased the embryonic THF content of zebrafish (Chang *et al.*, 2014 ▶). In contrast, knockdown of FDH significantly decreased the THF level and increased the oxidative stress in embryos, but did not affect the embryonic 10-FTHF content (Hsiao *et al.*, 2014 ▶; Chang *et al.*, 2014 ▶). In the current study, we show that the structure of the bound THF is dramatically stabilized by zNt-FDH. We speculate that the additional THF binding site formed between the two zNt-FDH domains may also contribute to the antioxidative activity of FDH.

**Figure 9:**
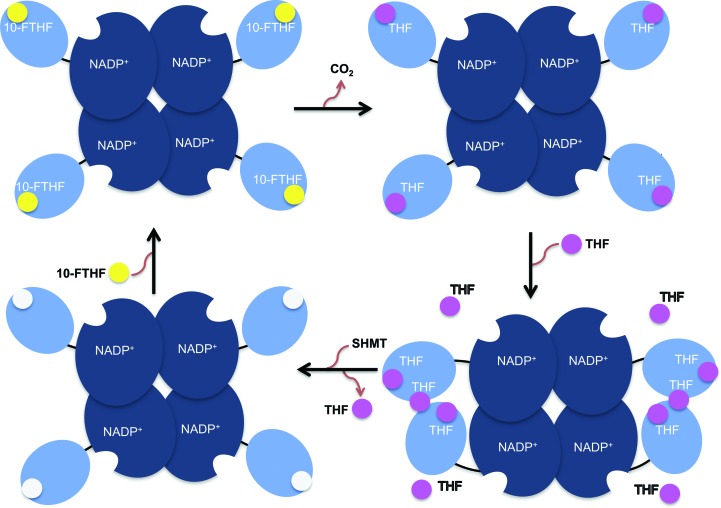
Proposed mechanism for the putative secondary THF binding at the interface between the two N-terminal domains in zebrafish FDH. The tetrameric FDH, with correctly oriented C-terminal NADP^+^-dependent dehydrogenase domains (blue) and N-terminal hydrolase domains (cyan), converts the formyl group of 10-formyl-THF into CO_2_ and generates THF. An intermediate domain between the N- and C-terminal domains is shown as a black line. The substrate (10-FTHF) and product (THF) are shown as yellow and pink spheres, respectively. Each Nt-FDH domain initially accommodates one substrate for catalysis to generate the product THF at the active site. THF subsequently accumulates in the proximity of the binding site as the reaction continues. The increased THF promotes the dimerization of Nt-FDH by binding to the interface between the two adjacent Nt-FDH domains, yielding an additional THF binding site. The presence of SHMT, which uses THF as its substrate, removes THF from FDH and hence eliminates product inhibition.

Our results suggest that as the catalytic reaction proceeds, the continuously accumulated THF might bind to the second THF binding site and prevent Tyr200 from switching back to the conformation allowing product release and substrate binding (Fig. 9 ▶). Upon attaining a particular level, increased THF in solution might also promote the dimerization of zNt-FDH by binding to the interface between the two zNt-FDH molecules. This is supported by DLS analysis, which shows that increasing the THF concentration in a zNt-FDH solution increases the molecular size of the zNt-FDH solution, and Western blot analysis, which shows that a major amount of dimers and a minor portion of oligomers of zNt-FDH are present in solutions with varied ratios of zNt-FDH and THF (Supplementary Fig. S4). In this scenario, there would be three THF binding sites (two active sites and one additional binding site) in a zNt-FDH dimer. Our studies provide structural evidence for the possible existence of an additional THF binding site, which might serve as a reference for FDH orthologues with a similar THF-binding stoichiometry, as well as an additional mechanistic explanation for inhibition of FDH production.

## Supplementary Material

PDB reference: zNt-FDH, 4ts4

PDB reference: complex with formate, 4r8v

PDB reference: complex with THF, 4qpd

PDB reference: complex with 10-FDDF, 4tt8

PDB reference: Y200A mutant, 4qpc

PDB reference: Y200A mutant, complex with 10-FDDF, 4tts

Supplementary Figures 1-4.. DOI: 10.1107/S1399004715002928/mh5163sup1.pdf

## Figures and Tables

**Table 1:** Statistics of diffraction data and structure refinement Values in parentheses are for the highest resolution shell.

	Native (PDB entry 4ts4)	Native + 10-FDDF (PDB entry 4tt8)	Native + THF (PDB entry 4qpd)	Native + formate (PDB entry 4r8v)	Y200A mutant (PDB entry 4qpc)	Y200A mutant + 10-FDDF (PDB entry 4tts)
Data collection						
Wavelength ()	1.000	1.000	1.000	1.000	1.000	1.000
Temperature (K)	110	110	110	110	110	110
Space group	*P*2_1_2_1_2	*P*2_1_2_1_2	*P*2_1_2_1_2_1_	*P*2_1_2_1_2	*P*2_1_2_1_2	*P*2_1_2_1_2
Unit-cell parameters ()						
*a*	104.67	104.08	54.5	103.98	104.46	103.64
*b*	53.88	52.69	100.42	51.98	53.96	54.38
*c*	60.63	60.5	122.52	59.94	60.88	61.01
Resolution ()	301.75 (1.811.75)	302.30 (2.382.30)	302.10 (2.182.10)	302.20 (2.282.20)	301.90 (1.971.90)	302.00 (2.072.00)
Completeness (%)	99.9 (99.9)	100 (100)	98.8 (89.0)	99.6 (99.9)	92.6 (99.8)	97.4 (100)
Multiplicity	6.9 (6.5)	5.6 (5.6)	5.2 (3.2)	5.5 (5.6)	5.6 (5.7)	6.0 (6.4)
*I*/(*I*)	33.8 (4.0)	15.8 (4.3)	18.9 (2.1)	20.5 (3.7)	14.1 (5.0)	12.6 (4.3)
*R*_merge_ † (%)	5.7 (44.0)	9.8 (43.9)	7.9 (43.7)	8.2 (48.6)	10 (35.2)	9.7 (33.2)
Refinement						
Resolution range ()	301.75	302.30	302.10	302.20	301.90	302.00
*R*_work_ ‡/*R*_free_ § (%)	18.0/20.7	18.0/22.4	18.5/23.3	19.3/23.6	20.4/22.5	20.1/24.5
No. of atoms						
Protein	2415	2415	4830	2415	2408	2408
Ligand/ion		48	110	24		34
Water molecules	332	162	432	85	345	291
*B* factors (^2^)						
Protein	30.1	32.8	34.3	39.8	30.0	20.9
Ligand/ion		45.8	40.6	57.4		37.8
Water molecules	37.0	34.2	36.7	36.7	36.3	25.0
R.m.s. deviations						
Bond lengths ()	0.007	0.004	0.006	0.007	0.006	0.008
Bond angles ()	1.300	0.968	1.010	1.114	1.053	1.260

† *R*_merge_ = 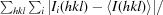 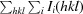, where *I* *_i_*(*hkl*) is the *i*th measurement and *I*(*hkl*) is the weighted mean of all measurements of *I*(*hkl*).

‡ *R*_work_ = 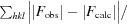 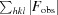, where *F*_obs_ and *F*_calc_ are the observed and calculated structure-factor amplitudes of reflection *hkl*, respectively.

§ *R*_free_ is calculated as for *R*_work_ but with a randomly chosen 5% of reflections that were omitted from refinement.

**(a):** 10-FDDF at the active site of wild-type zNt-FDH (PDB entry 4tt8) and the Y200A mutant (PDB entry 4tts).

		Wild type		Y200A mutant	
Substrate	Atom	P. atom	*D* ()	P. atom	*D* ()
10-FDDF	NA2	Ile90OH	3.01	Ile90OH	3.27
		Asp138OH	3.56	Asp138OH	3.20
	N3	Gly140OH	3.42	Asp138OH	3.14
				Gly140OH	3.23
	O4	Gly140OH	3.19	Gly140OH	3.13
		Asp142NH	3.05	Asp142NH	2.78
				Asp142OD1	3.59
		Water 6	3.17	Water 1	3.00
	OA1	His106ND1	2.68	Asp142OD2	2.74
		Asp142OD1	2.86	Water 84	3.54
		Asp142OD2	2.92		
	N	Ser87OH	2.88	Ser87OH	2.83
	O1			Ser87OH	2.81
	OE1	Arg60NE	3.48	Water 162	2.53
	OE2			Arg58NE	3.20
				Arg58NH2	2.09
				Arg60NH2	3.58
				Water 162	3.39

**(b):** THF at the active site of wild-type zNt-FDH (PDB entry 4qpd).

		Wild type (first molecule)		Wild type (second molecule)	
Product	Atom	P. atom	*D* ()	P. atom	*D* ()
THF	NA2	Ile90OH	3.03	Ile90OH	2.90
		Asp138OH	3.20	Asp138OH	3.22
	N3	Asp138OH	3.12	Asp138OH	3.18
		Gly140OH	2.98	Gly140OH	3.00
	O4	Gly140OH	3.19	Gly140OH	3.35
		Asp142NH	2.71	Asp142NH	2.79
		Water 2	2.88	Water 9	2.87
	N5	Asp142OD1	3.14	Asp142OD1	3.14
	N8	Gln88OH	2.88	Gln88OH	2.80
	N10	Asp142OD1	2.88	Asp142OD1	3.01
		Water 18	3.56		
	N			Tyr200OH	3.11
	O	Tyr200OH	3.32		
		Water 44	3.24		
		Water 24	3.11		
	O1	Water 160	3.57	Water 64	3.46
	OE1	Lys205NZ	2.44	Tyr200OH	3.12
				Ile203NH	3.30
	OE2			Water 64	3.44

**(c):** THF at the additional binding site of wild-type zNt-FDH (PDB entry 4qpd).

		Additional THF binding site	
Product	Atom	P. atom	*D* ()
THF	N3	Water 11	2.81
	N5	Water 118	2.93
	NA2	Gly282*B*OH	3.07
		Water 11	3.19
	O	Phe255NH	3.34
	O1	Water 188	2.55
	O4	Arg114HH21	2.28
		Water 118	2.91
	OE2	Leu278*B*OH	3.34

**Table 3:** Catalytic activities of wild-type zNt-FDH and related mutants

Protein	Relative activity† (%)
Wild type	100
F89A	16
R114A	22
Y200A	38
K205A	95

† The relative activity represents the ratio of the apparent *V*_max_ between the mutant and wild-type zNt-FDH. The apparent *V*_max_ was measured in a 1cm cuvette at 30C as described in [Sec sec2]2. The enzymatic reaction was initiated by adding purified zNt-FDH or mutant (15g) to the reaction mixture (0.8ml) in the presence of 20*M* 10-FTHF. The reported values are the average of the results obtained in at least three independent experiments.

**Table 4:** *R*_h_, MW and mass distribution of particles in zNt-FDHTHF solutions MW is the weight-averaged molar mass estimated based upon the particle conformation, size and density. Mass % is the estimated relative amount of mass (concentration) of each peak or species.

THF† (*M*)	*R*_h_ (nm)	MW (kDa)	Mass (%)
0 (1:0)	2.7	33	99.7
1 (1:0.02)	2.7	35	100
5 (1:0.1)	2.8	37	99.9
10 (1:0.2)	2.9	40	99.6
20 (1:0.4)	2.9	41	99.9
50 (1:1)	2.9	43	99.9
100 (1:2)	3.8	77	99.9
200 (1:4)	4.0	89	98.0
500 (1:10)	3.8	79	99.9
1000 (1:20)	3.9	81	97.8

† The ratios in parentheses indicate the corresponding ratios of zNt-FDH to THF.
