# Supplementary material for: Structures of the hydrolase domain of zebrafish 10-formyltetrahydrofolate dehydrogenase and its complexes reveal a complete set of key residues for hydrolysis and product inhibition
Source: Acta Crystallogr D Biol Crystallogr. 2015 Mar 27;71(Pt 4):1006–21. doi: 10.1107/S1399004715002928 (PMC4388273; doi:10.1107/S1399004715002928)
Supplement: Supplementary file 1 [file d-71-01006-sup1.pdf]

BIOLOGICAL
CRYSTALLOGRAPHY

Volume 71 (2015)

Supporting information for article:

Structures of the hydrolase domain of zebrafish 10-formyltetrahydrofolate dehydrogenase and its complexes reveal a complete set of key residues for hydrolysis and product inhibition

Chien-Chih Lin, Phimonphan Chuankhayan, Wen-Ni Chang, Tseng-Ting Kao, Hong-Hsiang Guan, Hoong-Kun Fun, Atsushi Nakagawa, Tzu-Fun Fu and Chun-Jung Chen

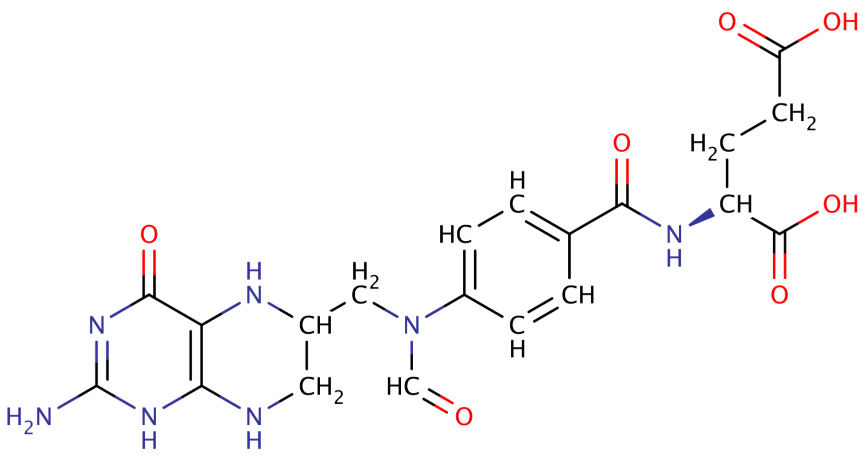

10-formyltetrahydrofolate (10-FTHF)

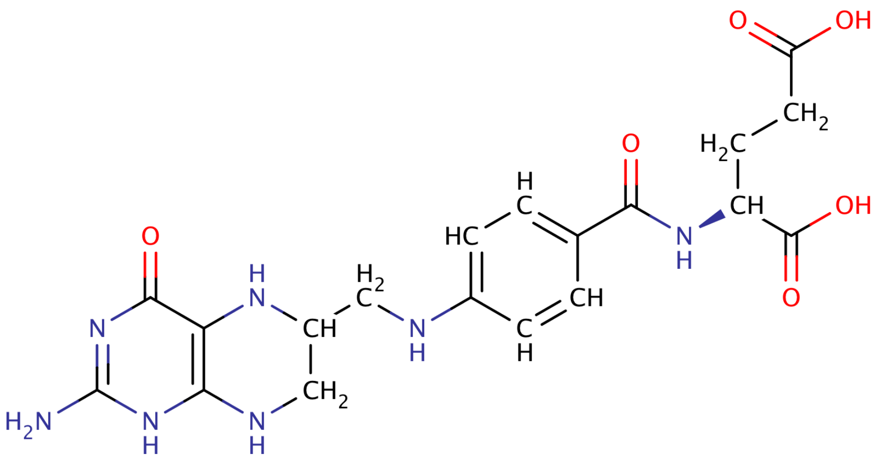

tetrahydrofolate (THF)

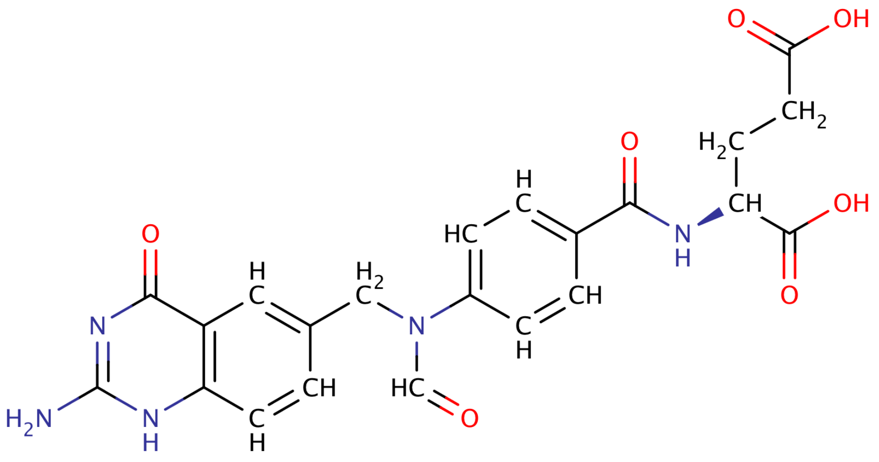

10-formyl-5,8-dideazafolate (10-FDDF)

Figure S1. Structures of 10-formyltetrahydrofolate, 10-formyl-5,8-dideazafolate and tetrahydrofolate.

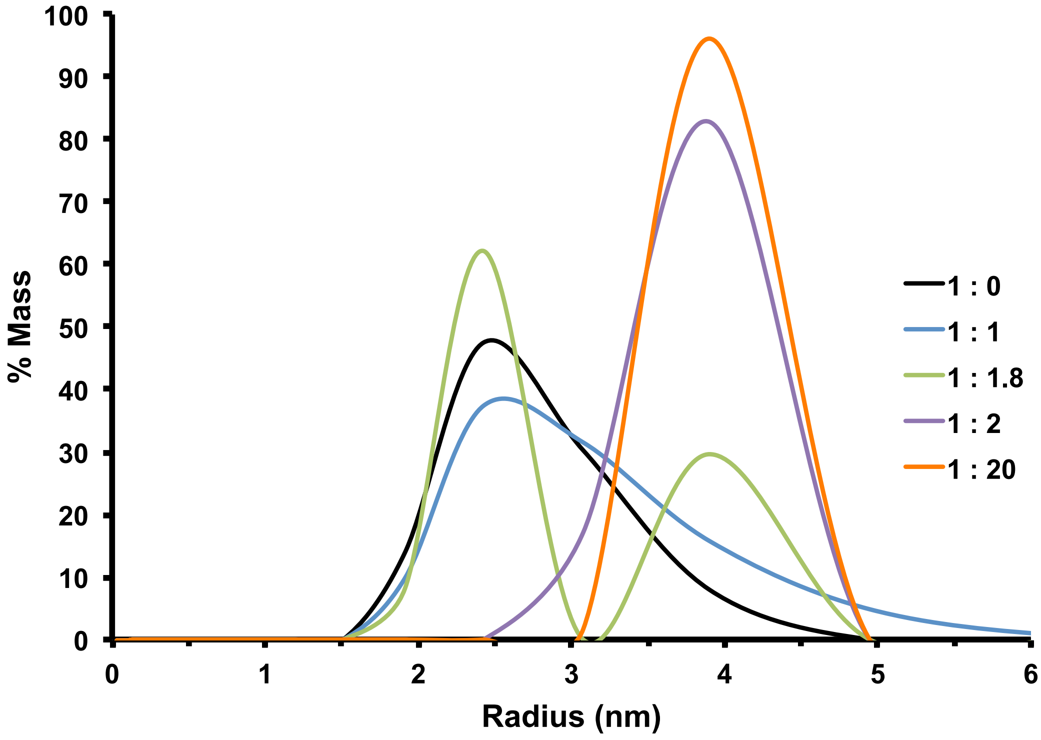

Figure S2. The transition state of zNt-FDH monomer and partially dimer from molar ration 1:1 to 1:2 in DLS. The profiles of THF to zNt-FDH at molar ratios of 1:0, 1:1, 1:1.8, 1:2 and 1:20 are colored in black, cyan, green, purple and orange, respectively.

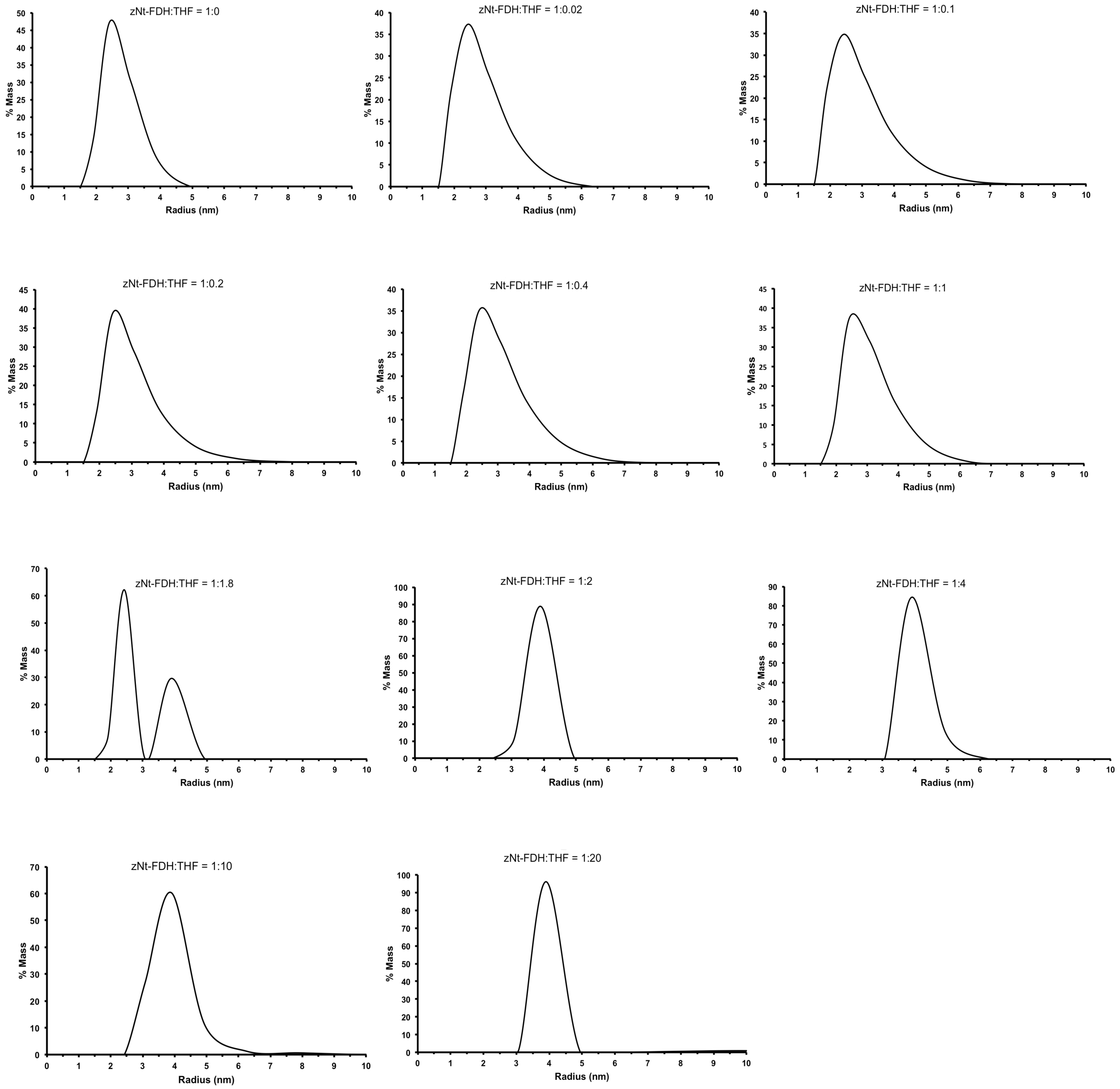

Figure S3. R_h and mass distribution of particles in the zNt-FDH-THF solutions. The zNt-FDH and THF molar ratios are varied from 1:0 to 1:20 in DLS.

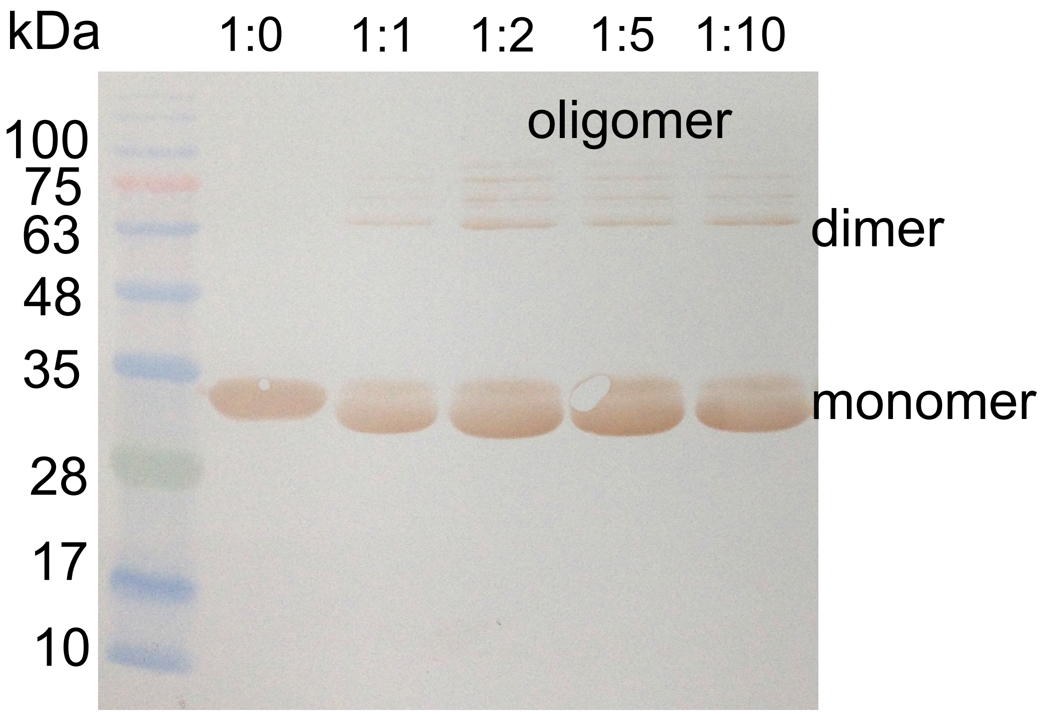

Figure S4. The Western blot analyses for zNt-FDH without (as a control) and with THF. The molar ratios of zNt-FDH and THF were varied from 1:0 to 1:10. The monomer, dimer or oligomer of zNt-FDH is observed on the PAGE.
